# Dynamic changes of region-specific cortical features and scalp-to-cortex distance: implications for transcranial current stimulation modeling

**DOI:** 10.1186/s12984-020-00764-5

**Published:** 2021-01-04

**Authors:** Hanna Lu, Jing Li, Li Zhang, Sandra Sau Man Chan, Linda Chiu Wa Lam, for the Open Access Series of Imaging Studies

**Affiliations:** 1 Department of Psychiatry, Multi-Centre, The Chinese University of Hong Kong, Tai Po Hospital, Hong Kong SAR, G/F China; 2 The Affiliated Brain Hospital of Guangzhou Medical University, Guangzhou, China; 3 Department of Mechanical and Automation Engineering, The Chinese University of Hong Kong, Hong Kong SAR, China

**Keywords:** Scalp-to-cortex distance, Cortical folding, Ageing, DLPFC, Brain stimulation, Modeling

## Abstract

**Background:**

Transcranial current stimulation in rehabilitation is a fast-growing field featured with computational and biophysical modeling. Cortical features and scalp-to-cortex distance (SCD) are key variables for determining the strength and distribution of the electric field, yet longitudinal studies able to capture these dynamic changes are missing. We sought to investigate and quantify the ageing effect on the morphometry and SCD of left primary motor cortex (M1) and dorsolateral prefrontal cortex (DLPFC) in normal ageing adults and mild cognitive impairment (MCI) converters.

**Methods:**

Baseline, 1-year and 3-year follow-up structural magnetic resonance imaging scans from normal ageing adults (n = 32), and MCI converters (n = 22) were drawn from the Open Access Series of Imaging Studies. We quantified the changes of the cortical features and SCDs of left M1 and DLPFC, including grey matter volume, white matter volume, cortical thickness, and folding. Head model was developed to simulate the impact of SCD on the electric field induced by transcranial current stimulation.

**Results:**

Pronounced ageing effect was found on the SCD of left DLPFC in MCI converters. The SCD change of left DLPFC from baseline to 3-year follow-up demonstrated better performance to discriminate MCI converters from normal ageing adults than the other morphometric measures. The strength of electric field was consequently decreased with SCD in MCI converters.

**Conclusion:**

Ageing has a prominent, but differential effect on the region-specific SCD and cortical features in older adults with cognitive impairments. Our findings suggest that SCD, cortical thickness, and folding of the targeted regions could be used as valuable imaging markers when conducting transcranial brain stimulation in individuals with brain atrophy.

## Introduction

Ageing is a complex nonlinear process that has negative impacts on the structures and functions of central neural system (CNS) [1]. Over the last decades, brain morphometry, derived from structural magnetic resonance imaging (sMRI), has emerged as a robust measure to quantify the cortical features [2, 3], which can enhance the accuracy of clinical diagnosis and prognosis, and further facilitate brain-based interventions in elderly population, such as in Alzheimer’s disease (AD) [4].

Advanced age is consistently associated with the reduction in total brain volume and cortical thickness [5, 6]; however, evidence regarding regional cortical changes measured using different scales is inconsistent [3]. For instance, the volumetric MRI-based measures of prefrontal cortex could discriminate the effect of age from the early morphometric changes resulting from neurodegeneration instead of global brain volume [7–9]. Meanwhile, studies that focused on surface-based measures, such as cortical thickness and folding, demonstrate more promising results in identifying the adults with mild cognitive impairment (MCI) from normal ageing adults and dementia patients [10]. Indeed, increasing evidence has confirmed that cortical regions exhibit ageing-related changes in morphometric features that are no larger than those measured across the whole cortex [11], implying a lack of anatomical specificity of progressive brain changes during normal ageing. Especially, the grey matter of lateral prefrontal cortex (PFC), as the vulnerable cortical region to AD pathology, has shown an inverted U-shaped across time [12], suggesting that global brain atrophy may not depict the regional brain changes of increased vulnerability due to pathological ageing.

Importantly, identifications of regional cortical features that are affected during ageing also emphasize on the key step toward developing personalized therapy for neurodegenerative diseases. Recently, transcranial current stimulation that delivers a small amount of electric current to the scalp and modulates the cortical activities is increasingly considered as a safe and effective intervention for individuals suffering from age-related brain diseases, such as late-life depression [13], MCI [14], and stroke [15]. Clinically, left primary motor cortex (M1) and left dorsolateral prefrontal cortex (DLPFC) are two commonly used therapeutic targets. The cortical features of the stimulation targets have been found to be related to the discrepancies in treatment outcome [16]. Except for cortical features, scalp-to-cortex distance (SCD), as an important parameter, could prominently influence the focality and strength of the electric field induced by brain stimulation and lead to heterogeneous treatment outcome [17–19]. For example, our previous studies have shown that older adults with dementia have an increased SCD of left M1 and DLPFC; therefore, same stimulation output may be insufficient to induce the desired therapeutic response in older adults with brain atrophy [20, 21]. Notably, when adjusting the stimulation output with the SCD of left M1, the SCD-adjusted output can induce effective stimulation intensity and improve the treatment response accordingly [22].

As mentioned, the links between region-specific cortical features, SCD, and electric field support the complex biophysical nature in ageing brains [20, 23, 24]. Considering the continuum of ageing to ageing-related neurodegenerative diseases [25], whether ageing has similar or differential effect on region-specific cortical features and SCD in individuals with increased risk of developing dementia is still unclear. Hence, the main purpose of this study was to determine the ageing effect on the cortical features and SCD of left M1 and DLPFC in normal ageing adults and mild cognitive impairment converters. A second objective was to examine and quantify the effect of SCD on the electric field through computational simulation model.

## Methods

### Participants

For detecting the cortical changes, study participants were drawn from the longitudinal brain dataset of the Open Access Series of Imaging Studies (OASIS) (https://www.oasis-brains.org) [26]. The cases who were dementia patients or had a history of major neurologic or psychiatric disorders or serious cerebrovascular conditions were excluded from this study. Overall, we included 32 normal ageing (NA) adults and 22 mild cognitive impairment (MCI) converters who had valid baseline and follow-up assessments. A three-wave longitudinal investigation of global cognitive function and structural magnetic resonance imaging was conducted at baseline, 1 year, and 3 years.

Global cognitive function measured by Mini-Mental State Examination (MMSE) was assessed by the physicians at the Washington University Alzheimer Disease Research Center (ADRC). All cases participated in accordance with the guidelines of the Washington University Human Studies Committee. Approval for public sharing of the anonymized data was also specifically obtained at the study site. The demographics, in terms of age, sex, and years of education and MMSE score of the participants, were directly obtained from the OASIS dataset.

### MRI data acquisition

Structural MRI (sMRI) data were acquired on a 1.5 T Vision scanner (Siemens, Erlangen, Germany) within a single session during which cushioning and a thermoplastic face mask were employed to minimize head movement. The T1-weighted magnetization prepared rapid gradient echo (MPRAGE) sequence was empirically optimized for grey–white matter contrast, with repetition time (TR) = 9.7 ms, echo time (TE) = 4.0 ms, inversion time = 20 ms, delay time = 200 ms, flip angle = 10**°**, resolution = 256 × 256 matrix (1 mm × 1 mm), slices = 128, and thickness = 1.25 mm.

### Surface-based morphometry

**Fig. 1:**
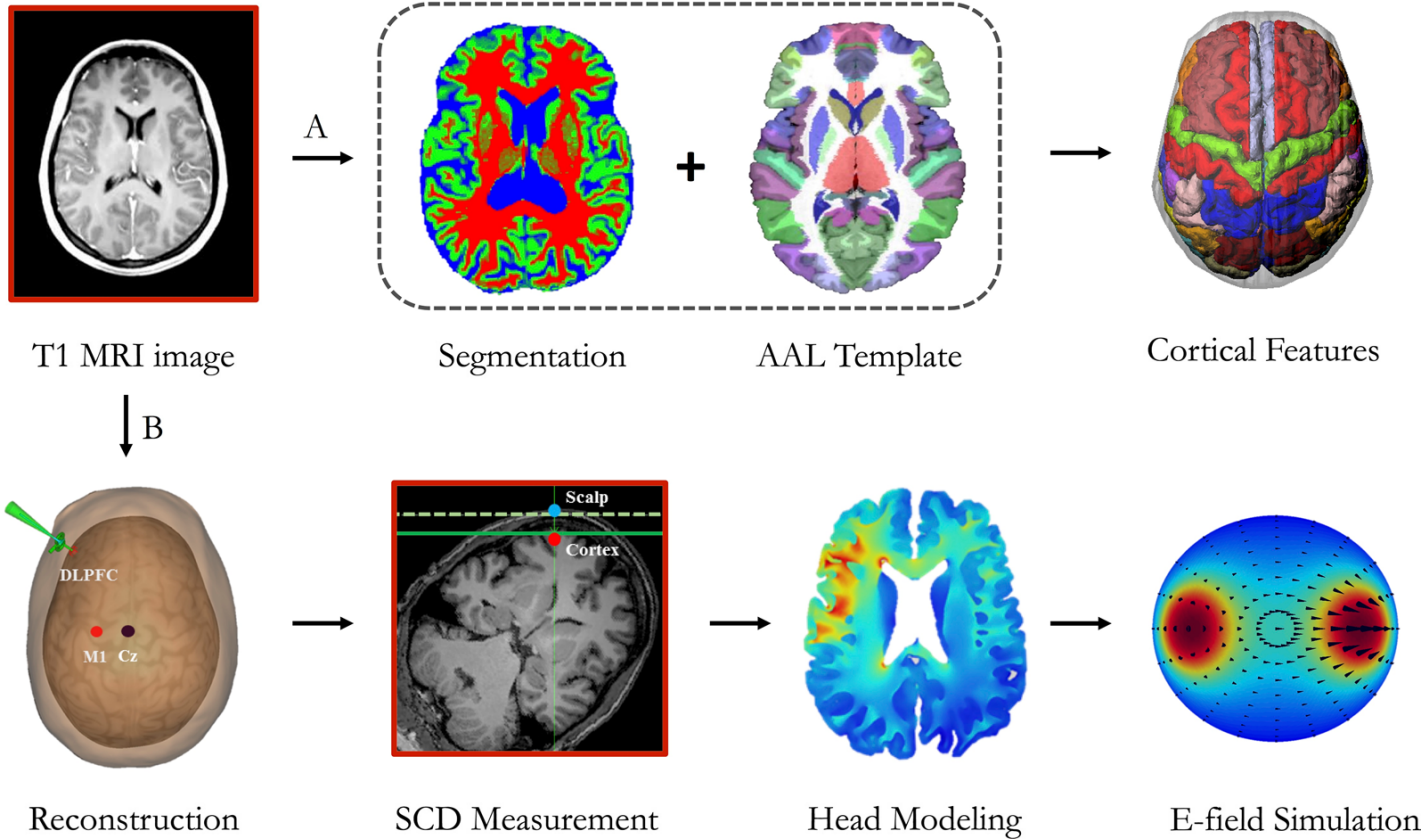
The pipeline of structural magnetic resonance imaging (sMRI) analysis: Path A represents the region-of-interest-based cortical mapping; Path B represents the measurement of scalp-to-cortex (SCD) and the construction of geometric simulation model

BrainSuite 16a (https://brainsuite.org/) was employed for surface-based morphometry (SBM) analysis of cortical features [27]. BrainSuite is an automatic cortical surface identification integrated package with the optimized version of Brain Surface Extraction (BSE), which is widely used in dementia and ageing research [28, 29]. Following the standardized pipeline (Fig. 1), the pre-processing of SBM includes: (1) motion correction; (2) intensity normalization; (3) removal of non-brain voxels; (4) segmentation into grey matter (GM), white matter (WM), and cerebrospinal fluid (CSF) images; (5) tessellation of the GM/WM boundary, and automated topology correction. At each step, we visually checked the outputs and manually corrected when there are segmentation errors (i.e., non-brain tissue).

### Volumetric measures

Based on the Automated Anatomical Labeling (AAL) template, the grey matter volume (GMV) of left M1 and left DLPFC was calculated individually. To adjust the heterogeneity of head size, Cendes method was used to correct the individual variance with total intracranial volume (TIV) through the formula [30]: corrected SV = (MBV × SV)/IBV. MBV is the mean brain volume in the group (a constant), SV is the regional grey matter volume, and IBV is the individual brain volume.

### Surface-based measures

Cortical thickness was calculated by computing the average distance (in millimetres) between pial surface and GM/WM boundary at each vertex on cortical surface. Cortical folding was measured by gyrification index (GI), a ratio of inner surface area to an outer surface area that smoothly encloses the cerebral cortex [31]. As part of SBM analysis, advanced processing steps for calculating cortical thickness and surface area, including atlas registration, spherical surface map, and parcellation, were performed based on AAL template. The cerebral cortex was parcellated into 68 anatomical regions for determining the region-specific mean cortical thickness and surface area. Each cortical map was visually inspected for proper registration and normalization prior to further analysis.

### Geometric measures

Region-specific scalp-to-cortex distance (SCD) was measured using the Brainsight neuronavigation system (https://www.rogue-research.com/tms/brainsight-tms/) (Rogue Research, Montreal, Canada). Based on individual sMRI data, we first conducted the 3D curvilinear reconstruction of scalp and cortex, and then adjusted the MRI-to-head co-registration using the anterior commissure-posterior commissure (AC-PC) line in the Montreal Neurological Institute (MNI) space. After co-registration, the locations of the targets on cortex were labeled with MNI coordinates (*x, y, z*) (Fig. 1).

**Fig. 2:**
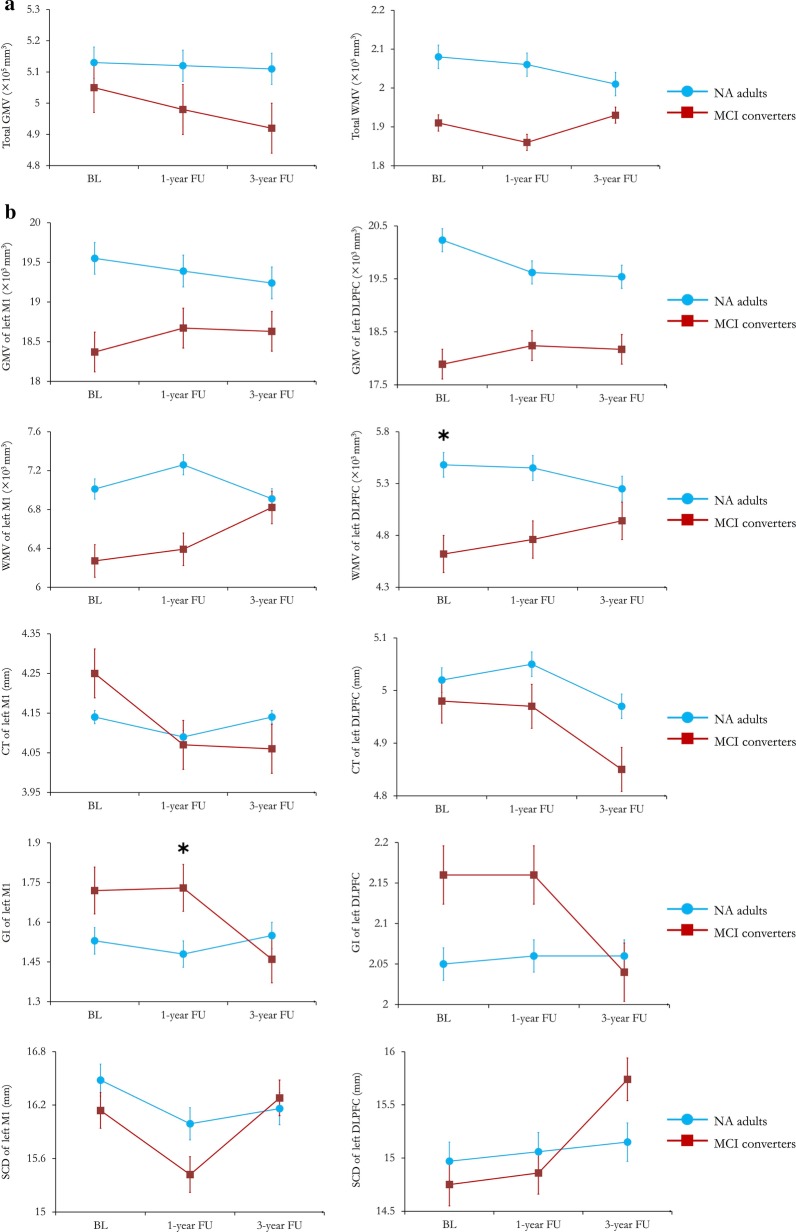
Trajectories demonstrating the region-specific morphometric measures listed in old adults with different cognitive statuses. In each plot, blue line represents the results from normal ageing (NA) adults, and red line represents the results from MCI converters. Error bars represent the standard error (SEM). * indicates significant between-group differences (*p* < 0.05)

Based on reconstructed cortex, we identified and pinpointed the locations of left M1 and left DLPFC individually (Fig. 2a). The hand representation of left M1 was determined according to the MNI coordinates as [x = − 42, y = − 16, z = 68] [20, 21, 32], representing as the “hook sign” on sagittal plane. The location of left M1 was verified within the grey matter on the top of paracentral gyrus. Regarding the conservative landmark approximating the cytoarchitectonic definition of prefrontal junction, we targeted left DLPFC with the MNI coordinates as [x = − 46, y = 45, z = 38] [16, 20]. The location of left DLPFC was verified within the grey matter on the top of middle frontal gyrus (MFG).

To better mimic the realistic brain stimulation, the corresponding locations of left M1 and DLPFC on the scalp were targeted in neuronavigation system by pointing the cursor to the scalp and then adjusting the orientation of the coil or electrode from the midline at 45**°**. Euclidean distance (*D*_*i*_), as a geometric index, was used to measure the distance between the two points locating on the scalp (*x*_*s*_*, y*_*s*_*, z*_*s*_) and the cortex (*x*_*c*_*, y*_*c*_*, z*_*c*_) with the following formula [20, 21, 33]:

*D*_*i*_ = $$\sqrt{{\left({x}_{s}-{x}_{c}\right)}^{2}+{\left({y}_{s}-{y}_{c}\right)}^{2}+{\left({z}_{s}-{z}_{c}\right)}^{2}}$$.

### Simulation of electric field

**Fig. 3:**
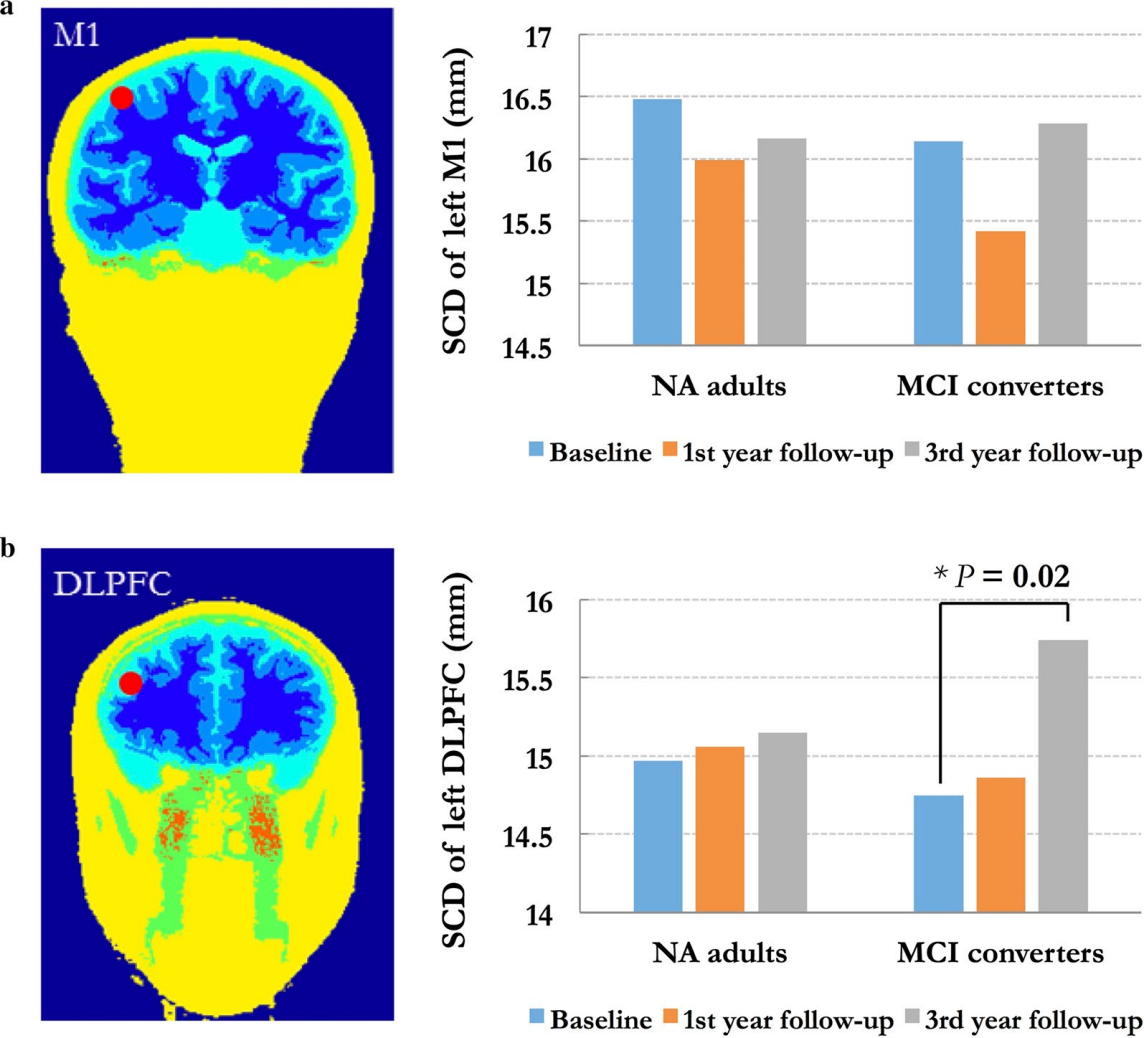
Demonstration of region-specific scalp-to-cortex distance (SCD) in normal ageing (NA) adults and mild cognitive impairment (MCI) converters. **a** Importing individual structural MRI data to the neuronavigation system and constructing the scalp and cerebral cortex; **b** localizing the targets and measuring the SCD; **c** comparing the ageing effect on the SCD of left primary motor cortex (M1) in NA adults and MCI converters; **d** comparing the ageing effect on the SCD of left dorsolateral prefrontal cortex (DLPFC) in NA adults and MCI converters

To examine the effect of SCD on the E-field in different therapeutic protocols, 3D head models were created by SPHERES (https://www.parralab.org/spheres/) and the realistic volumetric approach to simulate transcranial electric simulation (ROAST) toolbox (https://www.parralab.org/roast/) [34]. SPHERES is a stand-alone application that allows the considerations of arbitrary montages and the adjustments of brain parameters on a concentric sphere model by leveraging an analytical solution [35]. Regarding the most commonly used protocol in clinical practice and rehabilitation, we simulated the effect of SCD on the E-field induced by anodal tDCS over left DLPFC (i.e., F3 in international 10–20 system) (Fig. 3a).

To further quantify the effect of SCD on E-field, we calculated the entropy of the simulated E-field as described in information theory [36]. In brief, the entropy of the E-field induced by tDCS is quantified by calculating the pixel values within a two-dimensional centered region. Three geometric parameters of the grey-scale E-field include the following (Fig. 3c): represents the center of the E-field; represents the magnitude of the E-field; represents the edge of the E-field. The values of the three parameters were calculated and plotted using the Image Processing Toolbox embedded in MATLAB (https://www.mathworks.com/products/image.html).

### Statistical analysis

The differences of baseline cognitive performance and demographics in terms of age and sex were tested either with Chi-square (*χ*^2^) test for categorical variable or with independent sample *t* test for continuous variables. The comparisons of morphometric features were conducted using the code embedded in the BrainSuite 16a (https://neuroimage.usc.edu/neuro/Resources/BST_SVReg_Utilities). Multiple comparison correction was used by the above code using false discovery rate (FDR) estimation. Multilevel linear models were used to uncover the correlations from two levels: within group and between groups across different time points. Receiver-operating characteristic (ROC) analysis was used to evaluate the power of region-specific SCD in differentiating the seniors with different cognitive statuses. Pearson correlation coefficients were used to detect the relationship between morphometric measures, SCD and cognitive performance. The Chi-square test, pair-*t* test, multilevel linear models, and ROC analyses were performed using SPSS Statistics 24.0 (IBM, Armonk, NY).

## Results

### Ageing effect on cortical features

As shown in Table 1, age, sex, years of education, global cognitive function, mean cortical thickness, and total intracranial volume (TIV) were comparable between the two groups at baseline. MCI converters had worse performance on MMSE than NA adults at 1-year follow-up (*t* = 2.91, *p* = 0.009) and 3-year follow-up (*t* = 4.23, *p* < 0.001) (baseline vs. 3-year follow-up).

**Table 1:** Baseline demographics, cognitive function, and global cortical features

	NA adults (n = 32)	MCI converters (n = 22)	*t *(*χ*^2^)	*p* value
Age (years)	75.03 ± 8.12	75.84 ± 6.57	0.456	0.503
Sex (M/F)	15:17	11:11	0.324	0.572
Years of education	15.58 ± 2.91	14.01 ± 3.32	0.041	0.841
MMSE score	29.32 ± 0.71	28.68 ± 2.81	1.701	0.102
Mean CT	4.59 ± 0.36	4.59 ± 0.31	0.021	0.984
TIV (× 10^3^ mm^3^)	1518.52 ± 173.59	1483.84 ± 167.28	0.695	0.491

Data are raw scores and presented as mean ± SD

*MMSE* Mini-Mental State Examination, *CT* cortical thickness, *TIV* total intracranial volume

Within each group, no significant ageing effect was found in region-specific morphometric measures between the time points. Furthermore, we compared the cortical thickness, GMV, WMV, and cortical folding of left M1 and left DLPFC between NA adults and MCI converters (Table 2). Figure 2 representatively illustrates the trajectories of global and region-specific morphometric measures, of which cortical changes, particularly volumetric atrophy, were found in NA adults, but inverse changes of GMV and WMV were found in MCI converters. No cortical thinning was found in the left M1 in NA adults, but noticeable thinning was observed in the left M1 and left DLPFC in MCI converters. Meanwhile, nonlinear changes of the cortical folding of left M1 were also captured between the two groups over time. Compared to NA adults, MCI converters showed a prominent increase in the GI of left M1 at 1-year follow-up (*t* = 2.344, *p* = 0.024).

**Table 2:** Longitudinal changes of cortical features of left M1 and DLPFC

Morphometric measures	NA adults (n = 32)	MCI converters (n = 22)	*t* value	*p* value
Left M1				
GMV (×10^3^ mm^3^)				
Baseline	19.55 ± 2.58	18.37 ± 3.03	1.47	0.148
1st year FU	19.39 ± 2.35	18.67 ± 2.86	0.96	0.341
3rd year FU	19.24 ± 2.65	18.63 ± 3.49	0.71	0.487
WMV (×10^3^ mm^3^)				
Baseline	7.01 ± 2.02	6.27 ± 1.44	1.41	0.168
1st year FU	7.26 ± 1.98	6.39 ± 1.41	1.67	0.101
3rd year FU	6.91 ± 1.54	6.82 ± 1.71	0.17	0.861
Cortical thickness (mm)				
Baseline	4.14 ± 0.42	4.25 ± 0.55	− 0.841	0.405
1st year FU	4.09 ± 0.43	4.07 ± 0.43	0.171	0.865
3rd year FU	4.14 ± 0.51	4.06 ± 0.34	0.588	0.559
Gyrification Index (GI)				
Baseline	1.53 ± 0.26	1.72 ± 0.38	− 2.09	0.068
1st year FU	*1.48 ± 0.29*	*1.73 ± 0.51*	− *2.26*	*0.029*
3rd year FU	1.55 ± 0.35	1.46 ± 0.21	1.17	0.248
Left DLPFC				
GMV (×10^3^ mm^3^)				
Baseline	20.23 ± 4.01	17.89 ± 3.35	2.215	0.032
1st year FU	19.62 ± 3.14	18.24 ± 3.79	1.391	0.171
3rd year FU	19.54 ± 3.57	18.17 ± 4.27	1.218	0.229
WMV (×10^3^ mm^3^)				
Baseline	*5.48 ± 1.58*	*4.62 ± 1.05*	*2.325*	*0.024*
1st year FU	5.45 ± 1.65	4.76 ± 1.18	1.604	0.115
3rd year FU	5.25 ± 1.35	4.94 ± 1.49	0.764	0.449
Cortical thickness (mm)				
Baseline	5.02 ± 0.52	4.98 ± 0.72	0.236	0.815
1st year FU	5.05 ± 0.61	4.97 ± 0.41	0.357	0.722
3rd year FU	4.97 ± 0.41	4.85 ± 0.63	0.818	0.417
Gyrification Index (GI)				
Baseline	2.05 ± 0.38	2.16 ± 0.32	− 1.01	0.386
1st year FU	2.06 ± 0.41	2.16 ± 0.36	− 0.91	0.258
3rd year FU	2.06 ± 0.32	2.04 ± 0.25	0.32	0.737

Data are raw scores and presented as mean ± SD

*FU* follow-up, *M1* primary motor area, *DLPFC* dorsolateral prefrontal cortex, *GMV* grey matter volume, *WMV* white matter volume

### Ageing effect on geometric features

Table 3 demonstrates the changes of region-specific SCD across the follow-up observations. Overall, similar patterns of SCD changes were detected in both groups. No ageing effect was observed on the SCDs of left M1 and left DLPFC in NA adults, but significant ageing effect was found on the SCD of left DLPFC in MCI converters (Baseline vs. 3-year follow-up: *t* = − 2.54, *p* = 0.02) (Fig. 3b). Moreover, the SCD change of left DLPFC was correlated with the decreased score of MMSE (*r* = − 0.571, *p* = 0.011), and significantly contributed to the global cognitive decline (*R*^2^ = 0.326, *p* = 0.011, 95% CI: − 1.539, − 0.234).

**Table 3:** Longitudinal changes of scalp-to-cortex distance of left M1 and DLPFC

Geometric measures	NA adults (n = 32)	MCI converters (n = 22)	*t* value	*p* value
Left M1				
SCD (mm)				
Baseline	16.48 ± 3.01	16.14 ± 2.93	0.403	0.689
1st year FU	15.99 ± 2.66	15.42 ± 2.11	0.795	0.431
3rd year FU	16.16 ± 2.52	16.28 ± 3.21	− 0.144	0.886
Left DLPFC				
SCD (mm)				
Baseline	14.97 ± 2.98	14.75 ± 2.15	0.557	0.581
1st year FU	15.06 ± 2.62	14.86 ± 2.37	0.281	0.781
3rd year FU	15.15 ± 2.63	15.74 ± 2.03	− 1.855	0.083

### ROC analysis

To classify the groups with different cognitive statuses, the value of the area under the ROC curve (AUC) was used to test the discriminant power of the changes of cortical features and SCD. Neither MMSE nor volumetric measures showed a significant discriminative power; while, surface-based measures, the change of cortical folding (i.e., GI) of left M1 from baseline to 3-year follow-up had better performance to differentiate MCI converters from NA adults (AUC = 0.71, *p* = 0.014). Moreover, geometric measures, the SCD change of left DLPFC from baseline to 3-year follow-up also could discriminate MCI converters from NA adults (AUC = 0.687, *p* = 0.028).

### Simulation of SCD-associated E-field

Given the prominent effect of ageing on the SCD of left DLPFC in MCI converters, head models based on sMRI data were prepared for the E-field simulation of anodal tDCS by placing a rectangular 5 cm × 5 cm electrode on the scalp centered over left DLPFC (i.e., F3) (Fig. 3a). The SCD of left DLPFC was highlighted as a parameter of interest in SPHERES. In the head models, the isotropic conductivity values of the brain/non-brain tissues were adopted with default setting, including: 0.3 S/m for scalp, 0.03 S/m for skull, 2 S/m for CSF, and 0.03 S/m for cortical tissue [19–21].

**Fig. 4:**
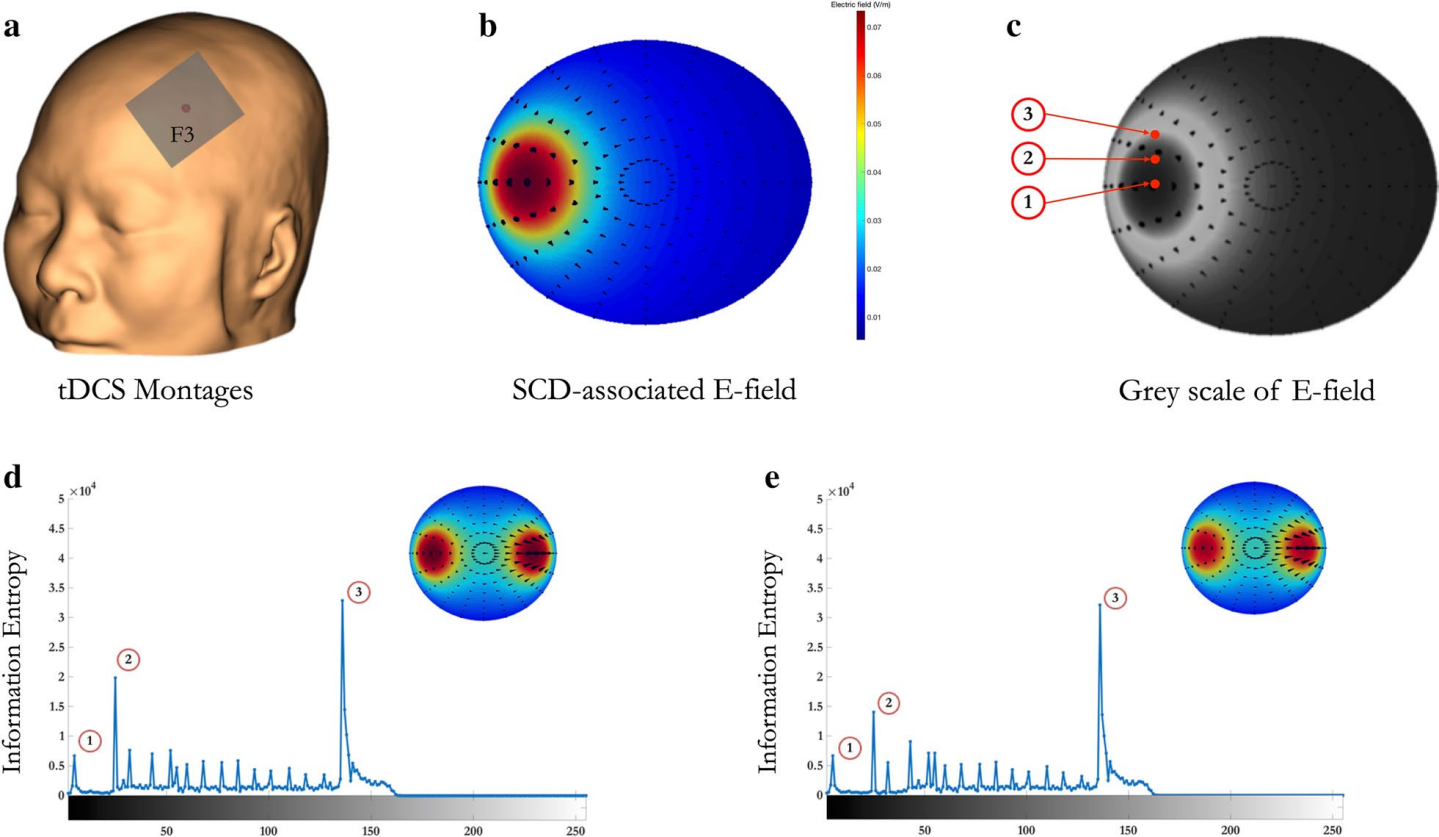
Three-dimensional geometric simulation model of anodal transcranial current stimulation. **a** We first reconstructed the scalp and placed anodal electrode over left DLPFC (i.e., F3 in the international 10/20 system); **b** second, completed the simulation of SCD-associated E-field using SPHERES and ROAST toolbox, and **c** transferred the E-field to grey-scale images for entropy analysis. Finally, we calculated the entropy changes of simulated E-field in MCI converters at baseline (**d**) and 3-year follow-up (**e**)

Using the SCD of left DLPFC as the “depth toward scalp” in SPHERES, the spatial distribution of the SCD-associated E-field was markedly decreased in MCI converters at 3-year follow-up. To quantify the effect SCD change on the E-field induced by tDCS, the information entropy of left DLPFC was 6.69 × 10^3^ at baseline (Fig. 4d) and 6.65 × 10^3^ at 3-year follow-up (Fig. 4e) (*t* = 4.17, *p* < 0.001). The magnitude of the E-field was 19.82 × 10^3^ at baseline and 14.02 × 10^3^ at 3-year follow-up (*t* = 13.6, *p* < 0.001). The entropy of the edge of the E-field was 32.84 × 10^3^ at baseline and 32.14 × 10^3^ at 3-year follow-up (*t* = 6.57, *p* < 0.001).

## Discussion

Using the MRI scans collected over a 3-year observational period, we examined and quantified the ageing effect on region-specific morphometric and geometric measures in older adults with different cognitive statuses. Generally, different from global measures, longitudinal volumetric, geometric, and surface-based measures showed nonlinear trajectories as represented by region-specific structural changes in normal ageing adults and MCI converters over time. In the context of global brain atrophy, ageing has a pronounced, but differential effect on regional cortical features, especially on cortical thickness, folding, and scalp-to-cortex distance (SCD). Moreover, the SCD change of left DLPFC has shown a significant impact on the electric field induced by transcranial current stimulation.

For decades, a majority of the cross-sectional studies has reported the structural brain changes with a linear pattern of age-related global decline [37]. Given the distributed nature of structural changes across the adulthood [38, 39], the marked atrophy in parenchymal volumes (i.e., grey matter and white matter) was mostly reported in older adults relative to younger adults [40]. Of note, even in late adulthood, a steeper and more rapid cortical atrophy was found in old–old adults (+ 90 years) compared to young–old adults (60–66 years) [41, 42]. Subsequently, longitudinal studies appeared to confirm these structural brain changes occurring at different rates for different cortical regions and tissue types [3], of which a greater annualized decline was found in total white matter volume rather than grey matter volume during normal ageing [43]. Indeed, a consistent pattern of brain atrophy in parenchymal volumes was observed in normal ageing adults, but an inversed pattern was detected in MCI converters during a 3-year period. Additionally, our results lead directly to the hypothesis that not all cortical regions demonstrate the similar patterns of volumetric brain changes [44]. In several targeted investigations, the most prominent regional reduction was observed in the fronto-parietal neocortex [45], particularly in the grey matter of prefrontal cortex [7, 46]. In accordance with these findings, regional grey matter loss over time was identical in left M1 and DLPFC among normal ageing adults compared to MCI converters, indicating that accelerated regional volume loss is not simply an effect of ageing on brain.

Whereas earlier studies measured cortical changes almost exclusively in terms of volumetric measures, more recent work adopted with surface-based measure reveals a more complex picture of ageing brain, with region-specific cortical differences demonstrating exclusive and often nonlinear trajectories [3]. Similar to regional volumetric changes, ageing-related cortical thinning was also mainly observed in the fronto-parietal cortex, including prefrontal cortex and motor cortex, in normal ageing adults and dementia patients [3, 7]. The nonlinear trajectories of cortical atrophy are normally represented with two morphometric features: (1) cortical thinning and (2) sulcal widening. In our observations, an overall accelerated cortical thinning was found in both left M1 and DLPFC. However, a region-specific pattern of cortical thinning was only detected in MCI converters during a mid-term follow-up period, of which the reduced cortical thickness is disproportionately greater in left M1 than left DLPFC at 1-year follow-up, but the cortical thickness was decreased rapidly in left DLPFC than left M1 at 3-year follow-up. During ageing, neuron loss and cortical atrophy may lead to cortical thinning [7], while decreased cortical folding might mainly reflect the underlying white matter change [47, 48]. Recent evidence highlighted that the age-related differences in the sulcal width of the frontal and central sulci were significantly associated with differences in the adjacent parenchymal volumes in older adults [49] and AD patients [50]. It seems that the sulcal morphometry measured by gyrification index (i.e., folding) is closely related to the cortical features in frontal and parietal cortex. Indeed, the anti-correlated trajectories of gyrification index and regional white matter volume observed in MCI converters also support this assumption. Moreover, the change of cortical folding, not white matter, showed the highest discriminative value in differentiating MCI converters from normal controls, indicating that the differences in cortical folding are progressive and could be detected and monitored during the long-term management of neurodegenerative diseases.

Combined with surface-based measures, scalp-to-cortex distance, as a geometric measure in the field of transcranial brain stimulation, is first studied to detect the accelerated structural brain changes in the region with a high degree of folding. Interestingly, the trajectories of SCD and cortical folding were nearly paralleled in normal ageing adults, but anti-correlated in MCI converters. The results may reflect the complicated dynamic changes in cortical features during pathological ageing. Different from other morphometric measures, significant ageing effect was only found in the SCD of left DLPFC in MCI converters, of which the SCD change of left DLPFC from baseline to 3-year follow-up also showed a modest power to discriminate MCI converters from normal ageing adults. In addition, with SCD as the key parameter of interest, we constructed the head model of anodal transcranial direct current stimulation (tDCS) over left DLPFC. Both strength and spatial distribution of the SCD-associated E-field were prominently decreased in MCI converters at 3-year follow-up. Therefore, the geometric simulation model demonstrates a novel and useful way to describe and quantify the complex biophysical interactions between SCD and E-field.

Moreover, SCD may essentially “scale-up” the collection of MRI-based morphometric measures (i.e., scalar variables) to serve as a vector in transcranial brain stimulation therapies. For example, despite cortical thickness and SCD share the same measurement scale (i.e., mm), SCD can provide additional geometric information from 3D space, even four-dimensional space (i.e., time dimension). The dynamic changes of region-specific SCD and the simulated E-field featured with quantitative measures may open the way for optimizing the technical parameters of brain stimulation, such as tDCS electrode montages, and TMS coil configurations, when conducting the brain-based interventions in age- or disease-specific populations.

### Implications for intervention

Understanding the trajectories of region-specific brain morphometry may allow us to harness the dynamic cortical changes to combat the cognitive decline during pathological ageing. Facilitating the scale-dependent cortical features, such as cortical thickness and scalp-to-cortex distance, shown to subserve brain-based interventions may expand the functional range of individuals contending with brain atrophy. Another potential application of MRI-based measures is to serve as an imaging marker to guide and inform the personalized rehabilitation and intervention.

### Limitations and future directions

The findings in this study should be interpreted with caution due to its limitations, of which the major one is the low number of participants. Nevertheless, it is somewhat remarkable that we found relatively robust ageing effect on cortical features, which further alludes to region-specific brain changes in the context of global brain atrophy. Furthermore, we were unable to examine the associations between morphometric measures and the domain-specific cognitive function during normal and pathological ageing in the present study due to the limitations of OASIS dataset.

Future research will aim to examine the cortical features and the functioning of core domains of cognition concurrently, so as to obtain a specific picture on the pattern of brain morphometry in different types of age-related neurodegenerative diseases, such as frontotemporal dementia (FTD), Alzheimer’s disease (AD), and Parkinson’s disease (PD). This will help to construct disease-specific brain atlas and further expand the applications of MRI-based measures in tracking the progression of cognitive decline and providing valuable information facilitated the personalized rehabilitation. Also, methodologies such as closed-loop transcranial brain stimulation with combined imaging modalities (e.g., TMS-EEG, tDCS-EEG) may represent the next frontier of neurotherapeutics in decoding the complex interlinks between ageing, brain morphometry, and disease progression.

## Conclusions

Structural MRI is a powerful non-invasive imaging modality that has enormous potential in studies of brain-based interventions. The longitudinal analyses of the region-specific cortical features in MCI converters help to shift towards greater awareness of nonlinear dynamic changes, indicating that the differential effect of ageing on cortical folding and scalp-to-cortex distance most likely occur in the course of pathological ageing. Furthermore, scalp-to-cortex distance demonstrates a promising and valuable geometric imaging marker for monitoring the cortical changes and optimizing the brain stimulation therapies for the individuals with brain atrophy.

## Data Availability

The dataset supporting the conclusions of this article can be found at the official website of the OASIS project: http://www.oasis-brains.org.

## Abbreviations

AAL: Automated Anatomical Labeling
AD: Alzheimer’s disease
AUC: Area under the ROC curve
BSE: Brain surface extraction
CNS: Central neural system
DLPFC: Dorsolateral prefrontal cortex
GI: Gyrification index
GMV: Grey matter volume
M1: Primary motor cortex
MCI: Mild cognitive impairment
MMSE: Mini-Mental State Examination
MRI: Magnetic resonance imaging
NA: Normal ageing
OASIS: Open Access Series of Imaging Studies
PFC: Prefrontal cortex
ROC: Receiver-operating characteristic
SBM: Surface-based morphometry
SCD: Scalp-to-cortex distance
TDCS: Transcranial direct current stimulation
TIV: Total intracranial volume
TMS: Transcranial magnetic stimulation
WMV: White matter volume
